# Impact of Digital Economy on the Provision Efficiency for Public Health Services: Empirical Study of 31 Provinces in China

**DOI:** 10.3390/ijerph19105978

**Published:** 2022-05-14

**Authors:** Yuwen Lyu, Yuqing Peng, Hejian Liu, Ji-Jen Hwang

**Affiliations:** 1 School of Economics and Statistics, Guangzhou University, Guangzhou 511400, China; 1111764007@e.gzhu.edu.cn; 2 School of Journalism and Communication, Guangzhou University, Guangzhou 511400, China; 3 Institude of Communication Studies, Communication University of China, Beijing 100024, China; 4 School of Education, Guangzhou University, Guangzhou 511400, China; 5 School of Policy and Government, George Mason University, Arlington, VA 20301, USA; jhwang29@gmu.edu; 6 Institute for Global Public Affairs Research, Bethesda, MD 20817, USA

**Keywords:** digital economy, digital government, government performance, regulatory quality, public health provision efficiency

## Abstract

The digital economy is booming in China and has become the world’s largest after the United States’. Since China entered the era of the digital economy, its digital technology has radiated into various fields. This study is to examine the impact of China’s digital economy on the provision efficiency of public health institutions and the mechanism of action between them. Specifically, it measures the development level of China’s digital economy, and the provision efficiency of public health institutions from 2009 to 2018. The research also explores the relationship between China’s digital economy and its provision efficiency, through the Tobit-DEA model. An analysis of the regional heterogeneity indicated that the performance of China’s digital economy in the eastern region has a significant positive effect on improving the efficiency of the public health sector. This further confirms that the digital economy has strengthened China’s ability to deal with public health crises during the COVID-19 pandemic. A further mediation effect analysis showed that China’s digital economy optimizes the efficiency of public health provision by improving governmental performance and regulatory quality. This shows that the development of the digital economy promotes the construction of digital government, and thus improves the quality of governmental supervision and governmental performance, which has a significant positive effect on the efficiency of the supply of public health services. During the COVID-19 pandemic especially, government delivery of public health services was critical in addressing public health crises. Therefore, based on the results of our empirical analysis, this study provides policy suggestions for improving the efficiency of public health service provision in the era of the digital economy.

## 1. Introduction

The digital economy is a new economic development mode, with data as the production factor and digital technology as the carrier. There are many signs that something new has happened to the American economy in the past decade. The digitalization of information, combined with the internet, represents a generic form of technology that is generating a vast array of new possible combinations, which we may refer to as the new economy for short [1]. Digitization is generating great interest around the world. This shift is horizontal and involves all sectors of economic activity. Abdelkhalek et al. proposed some of the impacts that digitalization may have on key sectors of the Moroccan economy and stated that the success of the digital transformation will affect the performance of the Moroccan economy in terms of employment, growth, and reductions in inequality [2]. Xu et al. showed that the digital economy was able to keep China’s economy stable during the COVID-19 pandemic [3].

The development of the digital economy not only contributes to the country’s economic growth, but also has a significant impact on public health. Digital technology is one of the accelerators of the global healthcare industry, and it is growing by a quarter every year. The future of healthcare may be adopting a new development model, characterized by the digitalization of medical data to help doctors make accurate medical decisions, and the use of mobile devices to monitor patients online and transmit medical indicators. Therefore, digital technology will likely affect the quality of public health services and the performance of national healthcare plans [4]. Jiang et al. used ARDL estimates to conduct empirical studies for specific countries, which showed that digitization would increase life expectancy in BRICS countries (excluding Brazil) in the long run [5]. Budd et al. found that the digital transformation of public health strengthened the global public health response to COVID-19, based on digital technologies such as the global network of mobile phones, large online data sets, connected devices, relatively low-cost computing resources, and algorithmic technologies such as machine learning and natural language processing. This is mainly reflected in population surveillance, case identification, contact tracing, and communication with the public based on mobile data [6].

During the COVID-19 pandemic, China’s public health response made an important contribution to the international response, saving many lives from the virus [7]. Digital technology played a crucial role in China’s approach to epidemic prevention and control [8]. Zhao’s empirical results show that there is a significant positive relationship between the development of the e-government and the digital economy [9]; it can be seen that, with the development of the digital economy, the construction of a digital government in China has significantly improved its public health response capability [10].

To sum up, to date, the abovementioned scholars have mainly focused on the impact of either digitalization or digital technology on public health but have not thoroughly studied the mechanism by which the digital economy specifically affects the efficiency of the provision of public health services. With the development of the digital economy, digital transformation will completely change the mode of the provision of public health services, further optimizing their efficiency. Therefore, in this study, we conducted a deep empirical analysis based on the predicted development of the digital economy and the efficiency of the provision of public services in 31 provinces in China from 2009 to 2018. On this basis, we further analyze the mediating effect of the mechanism between the two, with the aim of exploring the impact of the development of the digital economy on the service provision efficiency of public health and its mechanisms, so as to provide a basis for decision making and policy suggestions.

## 2. Statistical Indicators

### 2.1. Variable

#### 2.1.1. Indicators of Digital Economic Development Level

At present, the measurement of the digital economy development index is relatively difficult. The main reason for this is that there is still some controversy over the definition of the “digital economy” [11]. This stems from the continuous updating and iteration of the digital industry model, which leads to difficulties in the continuous and systematic tracking of output [12]. The establishment of an index system for the development level of the digital economy in this study refers to authoritative Chinese institutions and scholars, such as Bai Peiwen and Zhang Yun [13], approached via the four dimensions of digital industry, digital innovation, digital users and digital platform. Digital industry mainly includes the development degree of the basic industry of digital economy, digital innovation mainly reflects the level of intelligent technology, digital users mainly measure the digital level of users and the function of mobile payment of digital economy, and the digital platform mainly highlights the digital level of a network platform, so it can measure the level of development of the digital economy. In view of the availability of data, this study selected 2009–2018 data for measurement, and the specific indicator system summary is shown in Table 1.

#### 2.1.2. Indicators of the Efficiency of Public Health Service Provision

Since medical and health services are composed of medical care, health, and epidemic prevention services, this study referred to the work of Chu D. [14] and considered the availability of data to select the number of medical and health institutions/hospitals, the number of primary medical and health institutions, the number of health technicians, and the number of hospital beds as input indicators, while the number of working days of hospital beds, the utilization rate of hospital beds, the number of subsidized medical insurance and cooperative medical care policies, the number of individuals receiving direct medical assistance, the number of consultations, the number of admissions, the number of discharges, the average length of stay in hospital, and the emergency fatality rate (Countdown) were taken as the nine output indicators. The specific index system can be seen in Table 2.

#### 2.1.3. Exogenous Variables

Previous research by the scholars Xu [15] and Liu [16] indicated that the higher the population density (DENS) of a region, the more likely it will be that the public health services are provided by local governments to bring about economies of scale, thus improving the regional supply efficiency. The higher the education level of residents in the region (EDU), the stronger the civic consciousness will be, which will further enhance supervision by the government and promote the efficiency of supply. The intensity of household registration control (HR) and the urbanization rate (UR) will affect the epidemic among the population in the region, which will affect the activity of economic development, and thus affect the efficiency of the public service supply. Therefore, HR, DENS, EDU, BPSS, and UR were selected as control variables in this study.

#### 2.1.4. Mediation Variables

According to Zhao’s [17] research, the development of a digital economy improves the quality of government supervision (M1), corruption control (M2), government performance (M3), and the legal system (M4) through the construction of a digital government. Based on the theory of transaction cost and information asymmetry, the improvement of government supervision quality and government performance is beneficial to the establishment of an information transparency mechanism between the government and citizens, improving the degree of matching of the supply and demand of public health services, and improving the efficiency of service provision. The improvement of corruption control and the legal system can reduce the “rent-seeking behavior” of the government and public sector, thus reducing the cost of the supply of public health services and improving the efficiency of service provision. Therefore, the mediating variables of this study were selected as M1, M2, M3, and M4. The specific calculation method was primarily derived from the work of Zhao [17]; that is, government performance = number of public managers/total population at the end of the year; supervision quality = gross industrial production/total emissions of three forms of industrial waste; corruption control = rate of corruption, bribery, and malfeasance cases/number of public administrators; legal level = number of lawyers per 10,000 people.

### 2.2. Data Sources

The data analyzed in this study mainly came from the *China Urban Statistical Yearbook* (CUSY), the *China Statistical Yearbook* (CSY), the Digital Economy Industry Special Database (DEISD), the China Economic Information Center (CEIC), the *China Electronic Commerce Yearbook* (CECY), the *Statistical Report on Internet Development in China* (SRIDC), the Internet Information Center (IIC), and the *China Health Statistical Yearbook* (CHSY).

The specific information can be found in Table 3. The data sources for the variables selected in this study were the National Bureau of Statistics of China or other authoritative institutions, which could ensure the accuracy and authenticity of the data. In view of the lag of data updates and the availability of data, in this study we selected data from 2009 to 2018 as our research data.

### 2.3. Method

#### 2.3.1. Entropy Evaluation Method

In the field of economic statistics research, the entropy value method, as part of the comprehensive evaluation method, is a relatively scientific evaluation method. Authoritative objectivity is one of the advantages of the entropy method, mainly because the method was developed based on the information entropy theory. The difference between this method and subjective weighting is that the entropy method judges the weight of each indicator according to the degree of dispersion of the data, which avoids the randomness and imprecision caused by subjective weighting by researchers. The concept of “entropy” in the entropy method comes from thermodynamic theory. In information theory, information entropy can be judged by the discrete degree of information, so as to further determine the weight of each index. If the discrete degree of information data is large, its information entropy value is considered to be small; that is, the amount of information provided by the data is larger, so the weight is larger, and vice versa. Due to the objective weighting used in the entropy value method of the comprehensive evaluation method, the interference of subjective human factors can be reduced in the calculation of the results. Therefore, in this study, the comprehensive evaluation method was used in the selection of the calculation method for the digital economy development level index. The specific calculation steps of entropy evaluation method can be seen in Appendix B.

#### 2.3.2. Data Envelopment Analysis (DEA)

Regarding the measurement of efficiency, scholars mainly use the non-parametric method of data envelopment analysis (DEA). The DEA method was first created in the 1980s and is usually used to calculate multi-input and multi-output research objects. Since the measurement of the provision efficiency of public health services needs to measure the input and output and compare multiple research subjects, the DEA method was suitable. In the choice of measurement methods for supply efficiency, most relevant researchers again use data envelopment analysis (DEA). For example, Gunnar Rongen (1995) [18] measured the efficiency of fiscal expenditure by Norway’s public services and Husain and Abdullah (2000) [19] conducted a study of public sector service performance in Malaysia using this technique.

In terms of research methods, scholars have mainly used the BCC model, under the data envelopment analysis (DEA) framework, to measure the provision efficiency of public health services [20]. The BCC model was proposed by Banker, Charnes, and Cooper (1984) [21]. This method is based on the assumption that the input and output increase or decrease in the same proportion, which leads to deviations in the results. The super-efficiency SBM model is also one of the DEA models, and it has stronger explanatory power for efficiency evaluation results, due to the relaxation of the formal restriction of the stochastic frontier analysis function [22,23] Therefore, in this study we used the SBM super-efficiency model to calculate efficiency.

#### 2.3.3. Economic Model

In order to verify the impact of the digital economy on the efficiency of public health service provision, in this study we established an econometric model. The value of the service provision efficiency of public health services was between zero and two, so the explained variable belonged to the merged data type; that is, the restricted dependent variable was greater than zero. Therefore, maximum likelihood estimation (MLE) was more suitable for the estimation [24]. This model is called the “Tobit” model, and the specific model settings are as follows [25].

(1) $$BPHS\_efficiency_{it}=\beta_{0}+\beta_{1}DE_{it}+\sum_{j=2}^{6}\beta_{j}Control_{it}^{j}+\mu_{i}+\varepsilon_{it}$$

In the above Formula (1), *i* indicates the 31 provinces, and *t* indicates the year; the explained variable is the efficiency of the provision of public health services (*BPHS_efficiency_it_*) and the core explanatory variable is the digital economy development level (*DE_it_*). Each control variable is represented by $Control_{it}^{j}$, whereas the fixed effect is represented by $\mu_{i}$ and the random error term is denoted by $\varepsilon_{it}$.

For the model settings of the mediation effect, we referred to the recursive equation of Wen et al. [26] and built a mediation model (2) on the basis of the benchmark model (1):

(2) $$M_{kit}=\beta_{0}+\beta_{1}DE_{it}+\sum_{j=2}^{6}Control_{it}^{j}+\mu_{i}+\varepsilon_{it}$$

The settings of the comprehensive model (3) are as follows:

(3) $$BPHS\_efficiency=\beta_{0}+\beta_{1}DE_{it}+\beta_{2}M_{kit}+\sum_{j=3}^{7}\beta_{j}Control_{it}^{j}+\mu_{i}+\varepsilon_{it}$$

*M_kit_* in estimation model (2) and (3) is the mediating variable, representing the four mediating variables, namely, regulatory quality, corruption control, government performance and legal system level.

## 3. Results

This section is divided by subheadings and provides a concise and precise description of the experimental results, their interpretation, as well as the experimental conclusions that can be drawn.

### 3.1. Statistical Measurement Results

#### 3.1.1. Digital Economic Development

**Figure 1:**
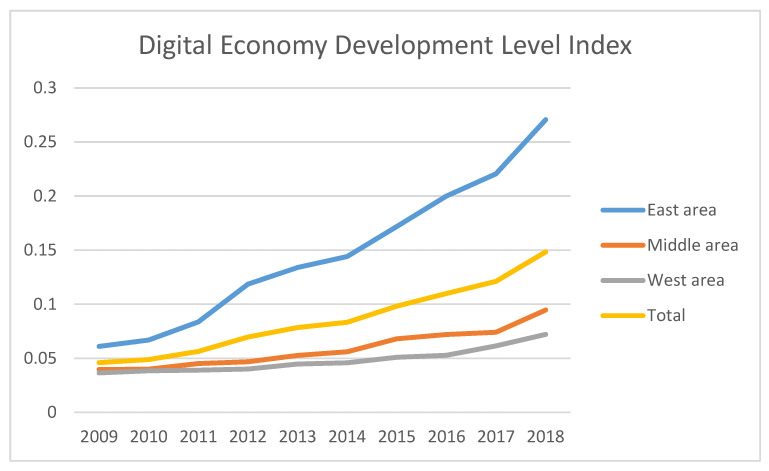
Mean value of the development level of digital economy in eastern, middle and western areas.

**Figure 2:**
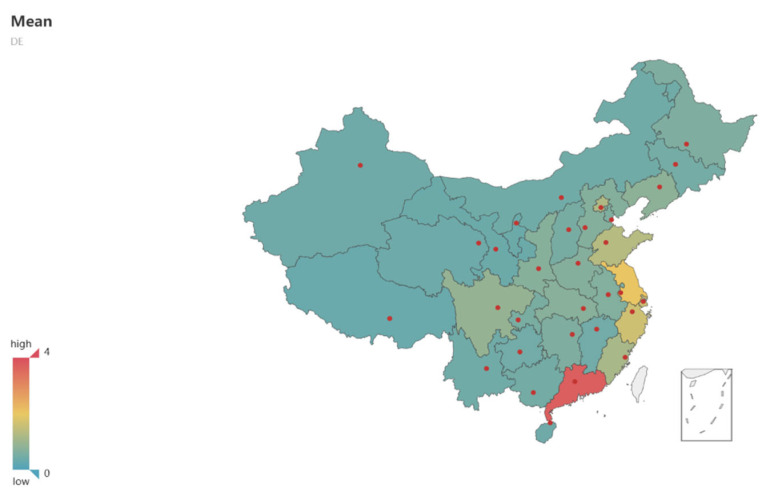
Geographical map of the mean value of digital economic development in the 31 provinces of China from 2009 to 2018.

Before measuring the development level of the digital economy, it is necessary to preprocess the data of the three-level indicators. For data with missing values, if the index is in line with or near to normal distribution, the mean filling method is used to fill in the missing values; otherwise, the median method is used. Based on the index system constructed above, the digital economy development level index for 31 provinces in China from 2009 to 2018 was calculated by the entropy method, denoted as DE. The calculation results can be seen in Table A2, while the mean development level of the digital economy in the eastern and central regions can be seen in Table 4 and Figure 1. Among them, China is divided into three economic belts, namely eastern region, central region and western region. The specific classification can be seen in Table A1.The geographical map of the mean development level of the digital economy in the 31 provinces can be seen in Figure 2.

#### 3.1.2. Efficiency of Public Health Service Provision

**Figure 3:**
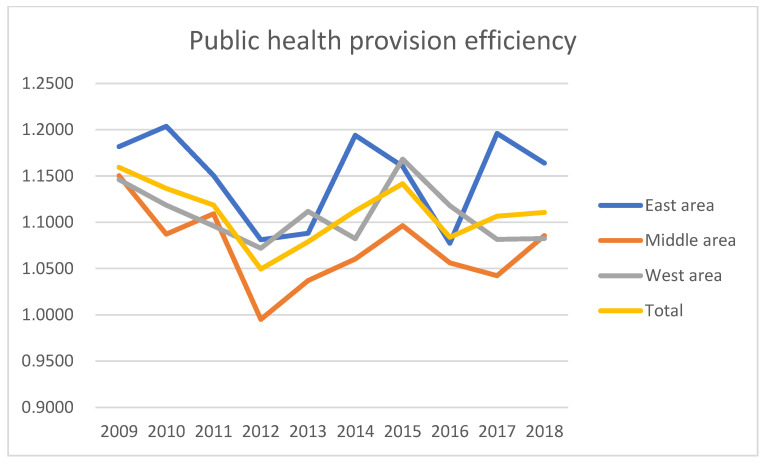
Mean value of the public health provision service efficiency in eastern, middle and western areas.

**Figure 4:**
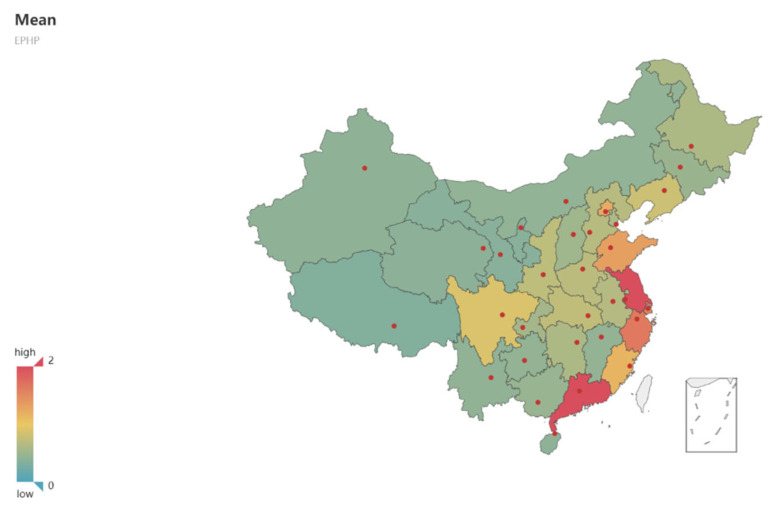
Geographic map of the mean value of public health service provision efficiency in 31 provinces in China from 2009 to 2018.

According to the input–output indicators of public health provision efficiency evaluation, shown in Table 2, this study uses the super-efficiency algorithm of the pyDEA (python for data envelopment analysis) package in the open-source software Python to measure the efficiency value. The measurement results can be seen in Table A3, the average values of the eastern, central and western regions can be seen in Table 5 and Figure 3, and the geographic map of the average value of digital economy development in China’s 31 provinces can be found in Figure 4.

### 3.2. Estimation Results

The results of the descriptive statistics of the variables show that the development of the digital economy affects the efficiency of public health service provision, as can be seen in Table 6. The core explanatory variable selected in this study is the development level of the digital economy, and the explained variable is the public health provision efficiency. The control variables are household registration control (HR), population density (DENS), national education level (EDU), general public service expenditure (BPSS) and urbanization rate (UR).

It can be seen from the correlation test results in Table 7 that the correlation coefficient between variables is low; the correlation coefficient between national education level (EDU) and general public service expenditure (BPSS) is the highest, at 0.4463.

Before model estimation, the variance inflation factor is calculated for the variables to determine whether there is multi-collinearity. The maximum value of the VIF is 1.56, the minimum is 1.11, and the mean is 1.30. It is generally believed that the value of the variance inflation factor does not exceed 10; that is, there is no serious multi-collinearity problem, so the model can be estimated.

In the empirical analysis of the impact of the development of the digital economy on the efficiency of public health provision, this study uses the SBM-Tobit model. The estimation results are shown in Table 8.

### 3.3. Mediation Analysis Results

This study estimates the impact of the development level of the digital economy on the efficiency of public health provision in 31 provinces and cities in China from 2009 to 2018. The results show that only the eastern region experienced a significant impact. On this basis, this study constructs a mediating effect model in the eastern region to empirically test the impact of the development of the digital economy on the efficiency of public health provision and its mechanism. The specific results of the analysis of the mediation effect are shown in Table 9. The results of the mediation effect analysis show that the quality of supervision (M1) and government performance (M3) were important channels through which the digital economy affected the efficiency of public health provision; that is, digital development in the eastern region improved the quality and performance of regional government supervision, thereby improving the efficiency of public health service provision.

## 4. Discussion

### 4.1. Analysis of How Digital Economy Improves China’s Public Health Provision Efficiency

As can be seen from Table 2, the development level of China’s digital economy increased year by year from 2009 to 2018, both nationally and regionally. According to the results of the heterogeneous analysis, the mean value of the digital economic development level index in the eastern region is 0.1471, while that for the central region is 0.0589, and the western region’s is 0.0482. The central and western regions’ values are lower than the national average of 0.08603. The development of China’s digital economy is characterized by regional imbalance. As can be seen from the measurement results in Table 2, the provision efficiency of China’s public health service was fluctuating in this period. Overall, the provision efficiency of China’s public services can be characterized by complexity and regional characteristics. Therefore, to improve regional provision efficiency, one should not consider one single element alone. For example, one might increase the input scale of service provision, but also take comprehensive factors, such as regional and government political preference, into consideration.

An analysis of regional heterogeneity found that digital economy development in eastern China in the period had a positive effect on the efficiency of public health provision, while it had no significant effect in central and western China. This shows that the development of the digital economy was a priority in eastern China and had a significant impact on the provision efficiency of the public health service. This also fully demonstrates the capacity of eastern coastal cities to respond to the COVID-19 pandemic.

The core elements of digital economy include digital innovation, digital industry, digital users and digital platforms. Automation and artificial intelligence (AI) are enjoying renewed interest across multiple industrial sectors due to the impact of COVID-19 in 2019. Digital health technologies provide an important technical foundation for health management by providing a quantitative basis for drug trials, medical research, public health programs, pandemic responses, and the overall measurement of an individual’s health. During the pandemic, the US Food and Drug Administration (FDA) issued a number of interim policies to support digital health innovations, such as expanded guidance on digital therapy for mental illness and medical devices for remote patient monitoring [27]. Moreover, governments are increasingly using cloud computing to lower costs, increase access, improve quality and innovate in healthcare. With a population approaching 4.5 billion, Asia’s healthcare challenges massively limit economic growth and policy making. Using cloud computing in healthcare may help improve the quality of healthcare delivery and reduce the economic burden, enabling governments to effectively address healthcare challenges in a short time frame [28]. The global manufacturing base has been strongly impacted by labor shortages related to the spread of COVID-19 and is working to increase productivity by adopting digital manufacturing technologies that leverage artificial intelligence and the Internet of Things (IoT), which promise improved connectivity between provision chains. This trend can increase and unblock the flow of social capital, a potential resource in the provision chain that influences the provision chain performance of the healthcare industry [29]. Therefore, the digital economy may play an important role in promoting the efficiency of the provision of public health services. However, the digitalization of the medical industry involves problems, including data accuracy and the real-time security and privacy of digital users, which are all challenges that the digital medicine of the future will face [30]. The empirical analysis of regional data, as discussed earlier, can provide a means to solve those problems.

### 4.2. Mediation Analysis of How China’s Digital Economy Optimizes the Provision Efficiency of Public Health Services

Following the mediation test of the empirical dataset collected from the 31 provinces in China in the previous section, we can now consider the mechanism of China’s digital economy in order to understand how to improve China’s public health service provision efficiency. The quality of government supervision, corruption control, government performance and legal system level were adopted as the mediation variables in this study. In the analytical results of the mechanism test, it was found that the variables of government supervision quality and government performance have mediation effects, while the other two mediation variables have no significant effects. Through the mediation analysis, we found that the digital economy in eastern China increased the provision efficiency of public services by improving the supervision quality and performance of local governments. Combined with our analysis of existing policy reports [31], we found that most provinces and cities in the eastern region held a frontier consciousness of actively building a digital government in the period in question. According to the 2019 Digital Government Assessment report, China’s provincial governments, including in the Beijing, Shanghai, Zhejiang, Guangdong, Fujian, Sichuan and Guizhou provinces, are in the first stages of developing a digital government [32]. Eastern provinces account for 70% of all provinces in the first tier. Therefore, the eastern region not only has the innate advantage of strong digital foundations, but it also has the edge in terms of its big data governance concept. This further illustrated why only the eastern region exhibited a significant mediating effect on government supervision quality and government performance.

Franca et al. (2010) [33] found in an empirical study that information asymmetry affects the efficiency of non-profit organizations. Based on the theory of information asymmetry, it can be seen that the development of the digital economy drives the digital governance of local governments, and the improvement of government supervision quality can reduce the degree of information asymmetry between the government and citizens [34], thus improving the efficiency of supply. Based on the transaction cost theory [35], the improvement of government performance can help to reduce the cost of public health service supply, thus improving the efficiency of supply.

To sum up, the construction of a digital government plays a crucial role in the optimization of the efficiency of the provision of public services. It is necessary to strengthen the digital governing capability of local governments in order to optimize the efficiency of the provision of public health services in these areas.

### 4.3. Policy Recommendations for China’s Efficient Provision of Public Health in the Digital Era

According to the empirical results of this study, the digital effect brought about by the development of China’s digital economy is not only felt in the fields of the economy, employment, and people’s livelihood, but it also has a positive impact on the efficiency of the provision of public health services. Our mediation analysis demonstrated that the construction of digital governance has a significant mediating effect on the provision efficiency of public health services in these local governments. With the help of the efficient management of digital governance, the quality and performance of government supervision can further improve the efficiency of public health service provision. The efficient provision of public health services can help to control epidemics and improve the ability to tackle public health crises. Especially during the COVID-19 pandemic, digital governance plays an important role. According to the China Smart City Service Platform Development Report (2021) [36], the 31 local governments of the provincial-level administrative regions in China have increased the rate at which they are setting up epidemic prevention and controlled project sites by more than 60%. At the lower level, local governments have also constructed websites, providing a wide range of information retrieval services, such as online hospitals, infection path query functions, public opinion guidance, medical information query resolutions, health code explanations, regional risk level guidance, and so on [37].

Therefore, the policy implications of this study are as follows. First, it is necessary to strengthen the construction of institutional environment and narrow the development gap of the digital economy between regions. Based on the theory of the institutional environment, we know that institutions can effectively restrain the negative effects caused by the unbalanced development of the digital economy. Therefore, the government should strengthen the construction of the institutional environment for the supply of digital economy and public health services, reduce the influence of the “polarization effect” of digital industry agglomeration caused by the unbalanced development of the digital economy, and thus improve the efficiency of service provision. Second, it is necessary to promote the development of the digital economy in an all-round way to ensure the level of supply of public health services. The digital economy represents a new engine of economic growth for China. Through the development of the digital industry, the digital economy can effectively realize an upgrade in the traditional industry structure and help to address the “middle-income trap”, to achieve high-quality economic growth, to raise the scale of public finance expenditure, and improve the level of the supply of public health services and their efficiency. Third, it is important to promote the construction of a digital government across the board, especially in the central and western regions. The construction of a digital government is helpful to improve the quality of government supervision and government performance, and thus improve the efficiency of the provision of public services. In the era of the digital economy, the construction of government digital service platforms relying on artificial intelligence, blockchains, cloud computing, and other digital technologies can achieve the accurate matching of supply and demand of public services and ultimately achieve high efficiency in the provision of public health services.

Therefore, in the era of the digital economy, digital technologies, such as artificial intelligence, blockchains, and 5G, are rapidly changing the governance models of modern governments and the way of life of their people [38]. By building a digital governance system, governments can improve their quality and performance, and, thus, comprehensively optimize the efficiency of the provision of public health. At the same time, special attention should be paid to the problems of uneven regional development. It is necessary to strengthen the development of the digital economy in developing regions in order to fully optimize the efficiency of the provision of public health in China.

## 5. Conclusions

The main conclusions of this study are as follows. Firstly, according to the empirical analysis results, the high level of development of digital economy has a positive and significant impact on the efficiency of public health. Secondly, the result of the mediation analysis showed that the quality and performance of government regulations had a mediating effect. This means that China’s digital economy optimized the efficiency of the provision of public health by improving the quality and performance of government regulations. Therefore, it can be expected that public health will undergo a comprehensive digital transformation. In the context of the COVID-19 pandemic especially, the development of China’s digital economy can help to optimize its public health service provision efficiency and thereby reinforce its capacity for responding to public health crises.

However, this study has the following shortcomings. First, the measurement of the development level of the digital economy is still controversial, and the selection and timing of indicators in the indicator system in this paper also have certain limitations. It is our hope that more authoritative and time-updated data will be obtained in the future. Second, due to the availability of data, in this study we mainly analyzed the impact of the digital economy on the efficiency of the provision of public health services from the perspective of digital governance. Other mediating variables, such as public finance and the institutional environment, have not been discussed. It is our hope that future studies can analyze the mechanism of action between these variables in more detail. Third, the endogenous nature of the study must be noted. In this study, there were inevitably endogenous problems relating to the impact of the digital economy on the efficiency of the provision of public health services, which would affect the accuracy of the estimation results. We hope that future researchers can find reasonable instrumental variables in order to solve these problems.

## Figures and Tables

**Table 1:** Comprehensive evaluation index system of the digital economy development level.

First-Level Indicators	Second-Level Indicators	Third-Level Indicators	Unit
Digital economy development level	Digital industry	Proportion of employment in urban units in information transmission, computer services and software industries X1	%
		Software business revenue X2	Billion
		Proportion of information transmission, computer services and software industries in the total fixed assets of the society X3	%
		Digital HP financial index X4	/
	Digital innovation	Number of 5G industry authorized patents X5	PCS
		Number of industrial internet authorized patents X6	PCS
		Number of e-commerce authorized patents X7	PCS
	Digital users	Popularization rate of mobile telephones X8	PCS/one hundred
		Total telecommunications business X9	Billion
		Per capita internet broadband access users X10	PCS
	Digital platform	Number of domain names X11	Ten thousand PCS
		Number of internet users X12	Ten thousand person
		Number of websites X13	Ten thousand PCS

**Table 2:** Input and output indicators of public health provision efficiency evaluation.

First-Level Indicators	Second-Level Indicators	Second-Level Indicators	Unit
Public health service provision efficiency	Input indicators of public health service	Number of medical and health institutions_Hospitals	PCS
		Number of primary medical and health institutions	PCS
		Number of health technicians	PCS/Thousand people
		Number of beds in medical and health institutions	PCS/Thousand people
	Output indicators of public health services	Working days of hospital beds	Day
		Hospital bed utilization rate	%
		Number of people who are subsidized to participate in medical insurance and cooperative medical care	One hundred million people
		Number of those receiving direct medical assistance	Ten thousand people
		Number of consultations	One hundred million people
		Number of people hospitalized	Ten thousand people
		Number of people discharged	Ten thousand people
		Average length of stay in hospital	Day
		Emergency fatality rate (Countdown)	/

**Table 3:** Variables and Data Sources.

Variable Type	Research Subjects	Year	Data Source
Dependent Variable	31 provinces	2009–2018	CSY, DEISD, CEIC, SRIDC, IIC
Independent Variables	31 provinces	2009–2018	CUSY, CSY, CHSY
Control variables	31 provinces	2009–2018	CUSY, CSY
Mediation variable	31 provinces	2009–2018	CUSY, CSY

**Table 4:** Average development level of digital economy in eastern, central and western regions from 2009 to 2018.

Year	Mean Value Development Level of Digital Economy			
	East Area	Middle Area	West Area	Total
2009	0.061	0.0397	0.0365	0.0460
2010	0.0669	0.04	0.0384	0.0489
2011	0.0836	0.0452	0.039	0.0564
2012	0.1186	0.0468	0.0401	0.0697
2013	0.1339	0.0527	0.0447	0.0784
2014	0.1441	0.056	0.0458	0.0833
2015	0.1718	0.068	0.0509	0.0982
2016	0.1999	0.072	0.0528	0.1099
2017	0.2205	0.074	0.0614	0.1211
2018	0.2706	0.0947	0.0721	0.1484
Mean	0.1471	0.0589	0.0482	0.0860

**Table 5:** Average value of public health service provision efficiency in eastern, central and western regions from 2009 to 2018.

Year	Public Health Service Provision Efficiency			
	East Area	Middle Area	West Area	Total
2009	1.1817	1.1503	1.1461	1.1593
2010	1.2037	1.0872	1.1184	1.1364
2011	1.1505	1.1091	1.0960	1.1185
2012	1.0811	0.9951	1.0720	1.0494
2013	1.0880	1.0370	1.1117	1.0789
2014	1.1939	1.0603	1.0823	1.1122
2015	1.1604	1.0963	1.1683	1.1417
2016	1.0773	1.0562	1.1179	1.0838
2017	1.1960	1.0422	1.0815	1.1066
2018	1.1639	1.0855	1.0824	1.1106
Mean	1.1497	1.0719	1.1077	1.1097

**Table 6:** Descriptive statistics of variables.

Variable	Mean	SD	Min	Max	*N*
BPHS_efficiency	1.1133	0.2547	0.6522	1.9904	310
DE	0.0860	0.0893	0.0231	0.8133	310
HR	0.1510	0.2657	0.0304	1.6470	310
DENS	2781	1184	515	5821	310
EDU	14,100	9520	128	45,800	310
BPSS	414	467	46	7467	310
UR	0.5514	0.1464	0.1919	1.0562	310

**Table 7:** Correlation test results.

		DE	HR	DENS	EDU	BPSS	UR
BPHS_efficiency	1.0000						
DE	0.1256	1.0000					
HR	0.1414	−0.0886	1.0000				
DENS	−0.0184	−0.1438	−0.2035	1.0000			
EDU	0.0557	0.4154	−0.2054	0.0485	1.0000		
BPSS	−0.0609	0.1542	−0.0844	−0.0419	0.4463	1.0000	
UR	0.1310	0.2811	−0.3687	−0.0766	0.3188	0.2415	1.0000

**Table 8:** Tobit model estimation of the impact of the digital economy on public health efficiency.

	Total	East	Middle	West
BPHS_efficiency				
DE	0.2919	0.4138 **	−1.5218	1.9879
	(1.56)	(2.23)	(−1.28)	(1.31)
HR	0.2244 *	1.5275	−0.0220	0.1643
	(1.74)	(1.51)	(−0.02)	(1.19)
DENS	0.0000	0.0001 **	0.0000	−0.0000
	(0.39)	(2.57)	(0.30)	(−1.26)
EDU	0.0000	−0.0000	0.0000	0.0000
	(0.12)	(−0.58)	(0.80)	(0.13)
BPSS	−0.0000	−0.0000	0.0002	0.0002
	(−1.32)	(−1.25)	(0.88)	(0.72)
UR	0.3809 **	0.4654 *	−1.0546 **	0.1041
	(2.39)	(1.85)	(−2.24)	(0.26)
_cons	0.8319 ***	0.3331 *	1.4866 ***	0.9589 ***
	(6.99)	(1.68)	(4.14)	(3.52)
/				
sigma_u	0.1739 ***	0.0940 ***	0.1315 ***	0.1576 ***
	(7.16)	(2.70)	(3.38)	(3.85)
sigma_e	0.1713 ***	0.1781 ***	0.1154 ***	0.1929 ***
	(23.62)	(13.63)	(11.87)	(14.52)
*N*	310	110	80	120

*t* statistics in parentheses; *p* < 0.1 *, *p* < 0.05 **, *p* < 0.01 ***.

**Table 9:** Estimated results of mediation analysis.

	M1	BPHS _efficiency	M2	BPHS _efficiency	M3	BPHS _efficiency	M4	BPHS _efficiency
DE	−0.1005 **	0.5055 **	−0.0006	0.4037 **	0.4655 ***	0.3417 *	−4.7409 ***	0.4387 **
	(−5.02)	(2.39)	(−1.27)	(2.19)	(4.17)	(1.74)	(−4.21)	(2.31)
HR	−0.0500	1.6237 *	−0.0000	1.5560	−0.4147	1.6299	−9.6006	1.3737
	(−0.37)	(1.71)	(−0.00)	(1.58)	(−0.92)	(1.57)	(−1.22)	(1.32)
DENS	−0.0000 ***	0.0002 ***	0.0000	0.0002 ***	0.0000	0.0001 **	−0.0008 ***	0.0001 **
	(−5.50)	(3.01)	(0.43)	(2.63)	(0.83)	(2.55)	(−3.28)	(2.14)
EDU	−0.0000 *	−0.0000	0.0000	−0.0000	0.0000 ***	−0.0000	−0.0001 **	−0.0000
	(−1.96)	(−0.61)	(0.05)	(−0.55)	(3.86)	(−0.89)	(−2.45)	(−0.61)
BPSS	−0.0000 ***	−0.0000	0.0000	−0.0000	−0.0000 **	−0.0000	−0.0006 ***	−0.0000
	(−4.16)	(−0.99)	(0.82)	(−1.23)	(−2.12)	(−0.88)	(−6.94)	(−1.39)
UR	0.2395 ***	0.2842	−0.0030 ***	0.3965	0.2658 **	0.3833	4.3257 ***	0.6038 *
	(9.57)	(0.91)	(−4.92)	(1.39)	(2.28)	(1.49)	(3.39)	(1.81)
M		0.9538		−21.8241		0.1568		−0.0098
		(0.82)		(−0.47)		(1.23)		(−0.62)
_cons	0.0486 *	0.2728	0.0041 ***	0.4018 *	1.1747 ***	0.1772	5.5867 ***	0.3234
	(1.84)	(1.37)	(6.76)	(1.66)	(12.94)	(0.74)	(3.35)	(1.61)
sigma_u	0.0252 ***	0.0820 **	0.0004 ***	0.0885 **	0.0000	0.0978 ***	2.7349 ***	0.0941 ***
	(3.74)	(2.45)	(4.12)	(2.44)	(0.00)	(2.82)	(4.42)	(2.73)
sigma_e	0.0118 ***	0.1794 ***	0.0003 ***	0.1787 ***	0.1397 ***	0.1762 ***	0.5766 ***	0.1777 ***
	(13.69)	(13.61)	(13.94)	(13.48)	(14.83)	(13.67)	(13.98)	(13.65)
Sobel Test	0.31443492 ***				0.08811794 *		−0.07113303	
*N*	110	110	110	110	110	110	110	110

*t* statistics in parentheses; *p* < 0.1 *, *p* < 0 05 **, *p* < 0 01 ***.

## Data Availability

The data presented in this study are available on request from the corresponding author.

## Appendix A

**Table A1:** Division of the three regional economic belts.

Three Regional Economic Belts	
East Area	Beijing, Shanghai, Guangdong, Tianjin, Shandong, Liaoning, Jiangsu, Hebei, Zhejiang, Fujian and Hainan
Middle area	Anhui, Jiangxi, Shanxi, Jilin, Heilongjiang, Henan, Hubei and Hunan
West area	Guangxi, Neimenggu, Chongqing, Sichuan, Guizhou, Yunnan, Tibet, Shaanxi, Gansu, Qinghai, Ningxia and Xinjiang

**Table A2:** China’s digital economy development level for the 31 provinces from 2009 to 2018.

Economic Belts		2009	2010	2011	2012	2013	2014	2015	2016	2017	2018
East area	Beijing	0.0946	0.1051	0.1343	0.1913	0.2063	0.2153	0.2704	0.3263	0.3431	0.3744
	Tianjin	0.0386	0.0391	0.0456	0.0535	0.0545	0.0597	0.0665	0.0725	0.0819	0.1022
	Hebei	0.0459	0.0483	0.0545	0.0594	0.0704	0.0694	0.0747	0.0786	0.0728	0.0951
	Shanghai	0.0567	0.0628	0.0897	0.1228	0.1316	0.1493	0.1903	0.2274	0.2458	0.2961
	Jiangsu	0.0655	0.0767	0.0998	0.1609	0.1902	0.2092	0.2491	0.2926	0.3057	0.3792
	Zhejiang	0.0598	0.0682	0.0924	0.1528	0.1331	0.1489	0.1902	0.2230	0.2388	0.3281
	Fujian	0.0433	0.0479	0.0616	0.0787	0.0831	0.0944	0.1191	0.1643	0.2245	0.2371
	Liaoning	0.0397	0.0441	0.0536	0.0795	0.0913	0.0979	0.1054	0.0959	0.0940	0.0956
	Shandong	0.0515	0.0587	0.0702	0.1020	0.1721	0.1635	0.1681	0.1757	0.1610	0.1985
	Guangdong	0.1437	0.1534	0.1811	0.2700	0.3048	0.3401	0.4147	0.4996	0.6036	0.8133
	Hainan	0.0314	0.0321	0.0364	0.0340	0.0355	0.0376	0.0409	0.0425	0.0539	0.0568
	Mean	0.0610	0.0669	0.0836	0.1186	0.1339	0.1441	0.1718	0.1999	0.2205	0.2706
Middle area	Shanxi	0.0518	0.0446	0.0516	0.0480	0.0491	0.0486	0.0526	0.0508	0.0515	0.0710
	Anhui	0.0358	0.0377	0.0441	0.0468	0.0542	0.0590	0.0734	0.0831	0.0873	0.1262
	Jiangxi	0.0320	0.0345	0.0354	0.0358	0.0388	0.0405	0.0491	0.0488	0.0517	0.0750
	Henan	0.0420	0.0444	0.0498	0.0549	0.0651	0.0682	0.0794	0.0839	0.0812	0.1185
	Hubei	0.0373	0.0405	0.0488	0.0538	0.0643	0.0697	0.0916	0.0959	0.0996	0.1248
	Hunan	0.0366	0.0414	0.0438	0.0467	0.0479	0.0518	0.0588	0.0860	0.0862	0.1070
	Jilin	0.0402	0.0386	0.0438	0.0438	0.0456	0.0484	0.0491	0.0508	0.0572	0.0599
	Heilongjiang	0.0417	0.0379	0.0439	0.0449	0.0568	0.0619	0.0901	0.0765	0.0776	0.0750
	Mean	0.0397	0.0400	0.0452	0.0468	0.0527	0.0560	0.0680	0.0720	0.0740	0.0947
West area	Neimenggu	0.0392	0.0410	0.0423	0.0413	0.0446	0.0419	0.0433	0.0404	0.0427	0.0539
	Guangxi	0.0372	0.0381	0.0421	0.0404	0.0440	0.0464	0.0513	0.0507	0.0521	0.0690
	Chongqing	0.0313	0.0338	0.0397	0.0451	0.0482	0.0523	0.0606	0.0682	0.0806	0.0919
	Sichuan	0.0397	0.0450	0.0508	0.0681	0.0874	0.0912	0.1047	0.1181	0.1290	0.1623
	Guizhou	0.0392	0.0398	0.0389	0.0349	0.0379	0.0391	0.0413	0.0460	0.0518	0.0670
	Yunnan	0.0327	0.0325	0.0350	0.0336	0.0383	0.0380	0.0513	0.0475	0.0481	0.0632
	Shanxi	0.0465	0.0510	0.0540	0.0590	0.0657	0.0699	0.0808	0.0852	0.0892	0.1092
	Gansu	0.0336	0.0318	0.0328	0.0315	0.0338	0.0331	0.0359	0.0369	0.0434	0.0559
	Qinghai	0.0341	0.0367	0.0345	0.0341	0.0403	0.0392	0.0425	0.0400	0.0435	0.0449
	Xizang	0.0367	0.0396	0.0260	0.0258	0.0246	0.0251	0.0231	0.0234	0.0699	0.0387
	Ningxia	0.0335	0.0361	0.0317	0.0304	0.0315	0.0336	0.0324	0.0341	0.0442	0.0579
	Xinjiang	0.0337	0.0353	0.0401	0.0374	0.0405	0.0402	0.0434	0.0434	0.0423	0.0518
	Mean	0.0365	0.0384	0.0390	0.0401	0.0447	0.0458	0.0509	0.0528	0.0614	0.0721
Total	Mean	0.0460	0.0489	0.0564	0.0697	0.0784	0.0833	0.0982	0.1099	0.1211	0.1484

**Table A3:** The efficiency of the provision of public health services in China’s 31 provinces from 2009 to 2018.

Economic Belts		2009	2010	2011	2012	2013	2014	2015	2016	2017	2018
East area	Beijing	0.7074	1.4326	0.8035	0.9248	0.8760	0.8323	0.8751	0.8569	0.8521	0.8905
	Tianjin	1.6306	1.5317	1.5405	1.5519	1.2310	1.2943	1.2602	1.2020	1.9692	1.1405
	Hebei	1.5147	0.8261	1.3560	0.9603	0.9538	1.2005	1.0120	0.9800	0.9406	0.9971
	Shanghai	1.6180	1.9904	1.3201	1.1567	1.3058	1.8703	1.6508	1.0588	1.5461	1.9578
	Jiangsu	1.2359	1.1101	1.1247	1.0462	1.2094	1.4705	1.2853	1.3109	1.4207	1.4416
	Zhejiang	1.1224	1.0586	1.2038	1.0881	1.1159	1.1580	1.2997	1.0305	0.9642	1.0608
	Fujian	1.1988	1.0073	0.9734	1.0091	0.9606	0.9819	1.0372	1.0607	1.0396	1.1672
	Liaoning	0.7001	0.6810	0.7277	0.7249	0.7934	0.8856	0.9337	0.8929	0.8970	0.8893
	Shandong	0.9971	0.9808	1.0019	0.9001	0.8860	0.9137	0.9142	0.9752	0.9663	1.0161
	Guangdong	1.0482	1.2143	1.1852	1.4089	1.4071	1.4144	1.4604	1.4382	1.4886	1.1035
	Hainan	1.2252	1.4078	1.4184	1.1207	1.2294	1.1115	1.0361	1.0446	1.0712	1.1388
	Mean	1.1817	1.2037	1.1505	1.0811	1.0880	1.1939	1.1604	1.0773	1.1960	1.1639
Middle area	Shanxi	1.0110	0.6522	0.7784	0.7749	0.8497	0.8898	0.9621	0.9446	0.9936	1.0266
	Anhui	1.3346	1.4253	1.3267	1.0889	1.5275	1.2291	1.5366	1.5433	1.5198	1.3938
	Jiangxi	1.0677	1.1152	1.0929	0.9917	1.0613	1.1711	1.1748	1.1396	1.0047	1.0781
	Henan	1.0871	1.1385	1.2149	1.2834	1.1358	1.0820	1.0708	1.0606	1.0510	1.2702
	Hubei	1.1856	1.0110	1.1733	0.9111	0.9527	0.9920	0.9144	0.8958	0.8933	0.9581
	Hunan	1.2899	1.1225	0.9424	0.9502	1.0996	1.1840	1.1382	1.0533	1.0051	1.0600
	Jilin	0.8768	0.8733	0.8736	0.9771	0.7765	0.9254	0.9153	0.8418	0.8874	0.8764
	Heilongjiang	1.3495	1.3594	1.4709	0.9835	0.8930	1.0090	1.0583	0.9702	0.9824	1.0206
	Mean	1.1503	1.0872	1.1091	0.9951	1.0370	1.0603	1.0963	1.0562	1.0422	1.0855
West area	Neimenggu	0.9004	0.8504	0.8056	0.7923	0.8695	0.8512	0.8783	0.8793	0.8629	0.8735
	Guangxi	1.2038	1.3397	1.3557	1.0445	1.1745	1.1024	1.0865	0.9886	0.9803	1.1352
	Chongqing	1.4713	1.3539	1.3743	0.8857	1.0827	1.6362	1.7580	1.6899	1.4408	1.0438
	Sichuan	1.3497	1.1996	1.0348	0.8477	1.9606	1.2540	1.3706	1.4771	1.4213	1.3880
	Guizhou	1.2390	1.2436	1.2000	0.9167	1.0850	1.0003	1.9383	0.7851	0.7499	0.7998
	Yunnan	1.1200	1.1087	1.1198	1.0467	0.9929	1.0574	0.9887	1.5691	1.0407	1.1830
	Shanxi	0.8448	0.7621	0.7929	0.7947	0.7722	0.7452	0.7457	0.7278	0.7341	0.7798
	Gansu	1.0554	0.9033	0.9026	1.8066	0.9141	0.8903	0.9802	0.9757	1.2608	1.3613
	Qinghai	1.0151	1.0215	0.9805	0.8732	0.9789	0.8996	0.8768	1.0047	0.9714	0.9149
	Xizang	1.2023	1.3531	1.3157	1.3341	1.4336	1.3051	1.2421	1.2631	1.2296	1.1662
	Ningxia	1.2960	1.2879	1.2996	1.4469	1.2168	1.3793	1.2829	1.2778	1.4954	1.4290
	Xinjiang	1.0551	0.9974	0.9702	1.0748	0.8599	0.8661	0.8716	0.7770	0.7911	0.9139
	Mean	1.1461	1.1184	1.0960	1.0720	1.1117	1.0823	1.1683	1.1179	1.0815	1.0824
Total	Mean	0.0460	0.0489	0.0564	0.0697	0.0784	0.0833	0.0982	0.1099	0.1211	0.1484

## Appendix B

The steps involved in the calculation of the entropy evaluation method were as follows.

First, standardize the initial data; see Formula (A1) for the positive indicators and Formula (A2) for the negative indicators.

(A1) $$x_{ij}^{\prime }=\left(x_{ij}-\overset{¯}{x}\right)/s_{j}$$

(A2) $$x_{ij}^{\prime }=\left(\overset{¯}{x}-x_{ij}\right)/s_{j}$$

*x_it_* is the initial value of item *j* of sample *i*, *x_it_′* is the standardized value, $\overset{¯}{x}$ is the average value of item *j*, and *S_j_* is the standard deviation of item *j*. Since logarithms are used in the calculation process of the entropy method, standardized data cannot be used. In this study we solved this problem by carrying out the forward translation of the standardized data.

(A3) $$Z_{ij}=x_{ij}^{\prime }+A$$

$Z_{ij}$ is the value after translation, and *A* is the magnitude of translation, which was 0.01 in this study. Then, we calculate the weight by means of the formula presented in (A4).

(A4) $$p_{ij}=Z_{ij}/\sum_{i=1}^{n}Z_{ij}\left(i=1,2,\cdots ,n;j=1,2,\cdots ,m\right)$$

where *n* is 31, namely, the number of sample provinces and *M* is 13, i.e., the number of indicators of digital economic development level evaluation system. Then, we calculate the entropy value, according to the following formula.

(A5) $$\left(e_{j}\right):e_{j}=-k\sum_{i=1}^{n}p_{ij}\ln\left(p_{ij}\right)$$

(A6) $$k=1/\ln\left(n\right),e_{j}⩾0$$

The calculation of the difference values is achieved using the following formula.

(A7) $$\left(g_{j}\right):g_{j}=1-e_{j}$$

(A8) $$\left(w_{j}\right):w_{j}=g_{j}/\sum_{i=1}^{m}\left(j=1,2,\cdots ,m\right)$$

Finally, we calculate the 31 province’s digital economic development level index values, remembering to DE.

(A9) $$(F_{i}):F_{i}=\sum_{j=1}^{m}w_{j}p_{ij}$$

## References

[1] Carlsson B. (2004). The Digital Economy: What is new and what is not?. Struct. Change Econ. Dyn..

[2] Abdelkhalek T., Ajbilou A., Benayad M., Boccanfuso D., Savard L. How Can the Digital Economy Benefit Morocco and All Moroccans? In Proceedings of the 2021: Economic Research Forum (ERF). https://erf.org.eg/app/uploads/2021/11/1637566122_724_832622_1503.pdf.

[3] Xu A., Qian F., Pai C.-H., Yu N., Zhou P. (2022). The Impact of COVID-19 Epidemic on the Development of the Digital Economy of China—Based on the Data of 31 Provinces in China. Front. Public Health.

[4] Ter-Akopov G., Kosinova N., Knyazev S. (2019). Digital technologies in healthcare: Achievements and prospects. 1st International Scientific Conference “Modern Management Trends and the Digital Economy: From Regional Development to Global Economic Growth” (MTDE 2019).

[5] Jiang C., Chang H., Shahzad I. (2021). Digital Economy and Health: Does Green Technology Matter in BRICS Economies?. Front. Public Health.

[6] Budd J., Miller B.S., Manning E.M., Lampos V., Zhuang M., Edelstein M., Rees G., Emery V.C., Stevens M.M., Keegan N. (2020). Digital technologies in the public-health response to COVID-19. Nat. Med..

[7] Zhang S., Wang Z., Chang R., Wang H., Xu C., Yu X., Tsamlag L., Dong Y., Wang H., Cai Y. (2020). COVID-19 containment: China provides important lessons for global response. Front. Med..

[8] Pan X.-B. (2020). Application of personal-oriented digital technology in preventing transmission of COVID-19, China. Ir. J. Med. Sci. (1971-).

[9] Zhao F., Wallis J., Singh M. (2015). E-government development and the digital economy: A reciprocal relationship. Internet Res..

[10] Starr P. (2000). Health Care Reform And The New Economy: Does the new digital economy require a different vision for health reform—its principles as well as its possibility?. Health Aff..

[11] Zhang X. (2017). China’s digital economy development index and its application. Zhejiang Soc. Sci..

[12] Tao Z., Zhi Z., Shangkun L. (2020). Digital economy, entrepreneurial activity and quality development: Empirical evidence from Chinese cities. Manag. World.

[13] Peiwen Bai Y.Z. (2021). Digital economy, declining demographic dividend and the rights and interests of low-skilled workers. Econ. Res..

[14] Yin C.D., Han Y.D., Zhang T.B. (2015). Fiscal decentralization, public sector efficiency and health service supply. J. Financ. Econ..

[15] Jingqing Xu Y.Y. (2011). Empirical analysis of local Government basic public Service supply efficiency and its influencing factors: Based on modified DEA two-step method. Financ. Trade Res..

[16] Liu H. (2019). Household registration control, basic public service supply and urbanization: An empirical analysis based on urban characteristics and floating population monitoring data. Econ. Theory Bus. Manag..

[17] Zhao Y.T., Zhang Z., Feng T.W., Tao K.T. (2019). Big data development, institutional environment and government governance efficiency. Manag. World.

[18] Rongen G. (1995). Efficiency in the provision of local public goods in Norway. Eur. J. Political Econ..

[19] Husain N., Abdullah M., Kuman S. (2000). Evaluating public sector efficiency with data envelopment analysis (DEA): A case study in Road Transport Department, Selangor, Malaysia. Total Qual. Manag..

[20] Vitezic N., Segota A., Setnikar Cankar S. (2016). Measuring the efficiency of public health services by DEA. Int. Public Adm. Rev..

[21] Cooper W.W., Seiford L.M., Tone K. (2007). Data Envelopment Analysis: A Comprehensive Text with Models, Applications, References and DEA-Solver Software.

[22] Chang Y.-T., Park H.-S., Jeong J.-B., Lee J.-W. (2014). Evaluating economic and environmental efficiency of global airlines: A SBM-DEA approach. Transp. Res. Part D Transp. Environ..

[23] Li H., Fang K., Yang W., Wang D., Hong X. (2013). Regional environmental efficiency evaluation in China: Analysis based on the Super-SBM model with undesirable outputs. Math. Comput. Model..

[24] Shuai S., Fan Z. (2020). Modeling the role of environmental regulations in regional green economy efficiency of China: Empirical evidence from super efficiency DEA-Tobit model. J. Environ. Manag..

[25] Guo Y., Hu Y., Shi K., Bilan Y. (2020). Valuation of water resource green efficiency based on SBM–TOBIT panel model: Case study from Henan province, China. Sustainability.

[26] Wen Z.L., Zhang L., Hou J.T., Liu H.Y. (2004). Mediation effect test program and its application. Acta Psychol. Sin..

[27] Kadakia K., Patel B., Shah A. (2020). Advancing digital health: FDA innovation during COVID-19. NPJ Digit. Med..

[28] Raghavan A., Demircioglu M.A., Taeihagh A. (2021). Public health innovation through cloud adoption: A comparative analysis of drivers and barriers in Japan, South Korea, and Singapore. Int. J. Environ. Res. Public Health.

[29] Kim H.K., Lee C.W. (2021). Relationships among healthcare digitalization, social capital, and supply chain performance in the healthcare manufacturing industry. Int. J. Environ. Res. Public Health..

[30] Condry M.W., Quan X.I. (2021). Digital Health Innovation, Informatics Opportunity, and Challenges. IEEE Eng. Manag. Rev..

[31] Li J. (2021). Report on China’s Digital Government Construction.

[32] Hou H. (2017). The application of blockchain technology in E-government in China. Proceedings of the 2017 26th International Conference on Computer Communication and Networks (ICCCN).

[33] de França J.M.F., de Figueiredo J.N., Lapa J.d.S. (2010). A DEA methodology to evaluate the impact of information asymmetry on the efficiency of not-for-profit organizations with an application to higher education in Brazil. Ann. Oper. Res..

[34] Mann S., Wüstemann H. (2010). Public governance of information asymmetries—The gap between reality and economic theory. J. Socio-Econ..

[35] North D.C. (1990). A transaction cost theory of politics. J. Theor. Politics.

[36] Iresearch (2021). Report on the Development of China’s Smart City Service Platform.

[37] Wang W., Wu Z.G., Wang F. (2020). The impact of COVID-19 prevention and control on the construction of digital government and suggestions. Sci. Technol. Rev..

[38] Abdrakhmanova G., Vishnevsky K., Gokhberg L., Demidkina O., Demyanova A., Kovaleva G., Kotsemir M.N., Kuznetsova I.A., Ekaterina L., Ozerova O.K. (2021). Digital Economy. https://elibrary.ru/item.asp?id=46498727.

